# Application of the Enzymatic Electrochemical Biosensors for Monitoring Non-Competitive Inhibition of Enzyme Activity by Heavy Metals

**DOI:** 10.3390/s19132939

**Published:** 2019-07-03

**Authors:** Amir M. Ashrafi, Milan Sýs, Eliška Sedláčková, Amir Shaaban Farag, Vojtěch Adam, Jan Přibyl, Lukáš Richtera

**Affiliations:** 1 Department of Chemistry and Biochemistry, Mendel University in Brno, Zemedelska 1, CZ-613 00 Brno, Czech Republic; 2 Central European Institute of Technology, Brno University of Technology, Purkynova 123, CZ-612 00 Brno, Czech Republic; 3 CEITEC—Central European Institute of Technology, Mendel University in Brno, Zemedelska 1, CZ-613 00 Brno, Czech Republic; 4 Department of Analytical Chemistry, Faculty of Chemical Technology, University of Pardubice, Studentská 573, 532 10 Pardubice, Czech Republic; 5 Czech Republic CEITEC MU, Nanobiotechnol Group, Kamenice 5, CZ-62 500 Brno, Czech Republic

**Keywords:** glucose oxidase, heavy metals, amperometric biosensor, non-competitive inhibition

## Abstract

The inhibition effect of the selected heavy metals (Ag^+^, Cd^2+^, Cu^2+^, and Hg^2+^) on glucose oxidase (GOx) enzyme from *Aspergillus niger* (EC 1.1.3.4.) was studied using a new amperometric biosensor with an electrochemical transducer based on a glassy carbon electrode (GCE) covered with a thin layer of multi-wall carbon nanotubes (MWCNTs) incorporated with ruthenium(IV) oxide as a redox mediator. Direct adsorption of multi-wall carbon nanotubes (MWCNTs) and subsequent covering with Nafion^®^ layer was used for immobilization of Gox. The analytical figures of merit of the developed glucose (Glc) biosensor are sufficient for determination of Glc in body fluids in clinical analysis. From all tested heavy metals, mercury(II) has the highest inhibition effect. However, it is necessary to remember that cadmium and silver ions also significantly inhibit the catalytic activity of Gox. Therefore, the development of Gox biosensors for selective indirect determination of each heavy metal still represents a challenge in the field of bioelectroanalysis. It can be concluded that amperometric biosensors, differing in the utilized enzyme, could find their application in the toxicity studies of various poisons.

## 1. Introduction

Enzymes are organic catalysts produced within the living organisms. They speed up the biological reactions by lowering the activation energy. They can speed up the conversion of the substrate to the products in cellular metabolism up to 10 million times or more [1]. The conversion of the substrates by enzyme is highly specific. Many enzymes only show specificity for one substrate, while several structurally related substrates can be affected by another type of enzyme [2]. To initiate an enzyme-catalyzed reaction, the enzyme must bind to its substrate forming an enzyme–substrate complex [3]. Considering that the enzymes remain unchanged after the reactions, they catalyze and can be reused. Therefore, they are effective in a very small amount [4]. The enzyme catalyzes either the forward or backward reaction to the same extent [5]. However, the catalytic activity of an enzyme might be inhibited by an inhibitor. Enzyme inhibition is an important means by which the activity of enzymes is controlled. Inhibitors can be classified in different groups. In instances of cyanide and many nerve gases are considered as irreversible inhibitors or catalytic poisons as they completely deactivate the enzyme [6]. The competitive inhibitors have a similar shape to that of the substrate molecule. Thus, they are able to bind to the active site, preventing the binding of a substrate molecule [4]. On the other hand, a noncompetitive inhibitor interacts with the enzyme, but usually not at the active site. The noncompetitive inhibitor reacts either remotely from or very close to the active site [7]. Due to the strong binding capability between the heavy metals and sulfhydryl groups of proteins (non-competitive inhibition), they are also classified as enzymatic (catalytic) poisons [7,8]. This binding causes the structural changes and deteriorated enzymatic activities, which results in toxic effects of heavy metals at the whole organism level [9]. Hence, at sufficient concentration, the heavy metal ions such as Ag, Cd, Cu, and Hg are fatal to organisms or cause other adverse effects [10].

The reaction monitoring brings about the indispensable information about the molecular speciation [11] and provides key insights into reaction mechanisms [12], kinetics, and the biochemical process of the system. Moreover, since the enzymatic reactions are important for food [13], chemical [14], biofuel and medicinal fields [15], the real time monitoring of a reaction results in an enhancement of the efficiency and accuracy of the overall process. As an instance, in industries dealing with a bioprocess a quick test is required to gain insight into the activities of several biochemical compounds, such as enzymes to modify or further optimize the processes. Therefore, the monitoring of the enzymatic activity is in high demand. Various analytical methods were utilized for monitoring the enzymatic activity, including mass spectrometry [16,17,18], Raman spectroscopy [19], spectrophotometry [20], and the electrochemical techniques [21,22,23,24,25,26]. Considering their simplicity, the low cost and rapidness, the electrochemical techniques are preferred over other analytical techniques, which sometimes need a complicated pretreatment, filtration, and a well-skilled operator. The enzymatic sensors are developed by immobilization of an enzyme on the electrode and then applied for the concentration determination of the corresponding substrate. The main difference between enzyme-based biosensors is the immobilization procedure and the applied mediator [27]. In this work, an amperometric Glc biosensor was developed in which Gox, as the biorecognition element, was immobilized on the MWCNTs, and RuO_2_ was used as the mediator. Furthermore, the enzyme was covered by Nafion^®^ membrane in order to increase the stability of the sensor. The developed sensor was applied for the concentration determination of hydrogen peroxide and Glc. Moreover, the possibility of using the developed sensor as an electroanalytical approach to study the heavy metal toxicity (inhibition of enzymes activity) was investigated. The effect of the heavy metals cations (Ag^+^, Hg^2+^, and Cd^2+^) on the Gox enzyme activity was investigated. It must be mentioned that screening of enzyme activity of other enzymes is possible using the same principle as well.

## 2. Results and Discussion

### 2.1. Amperometric Transducer Design

**Figure 1:**
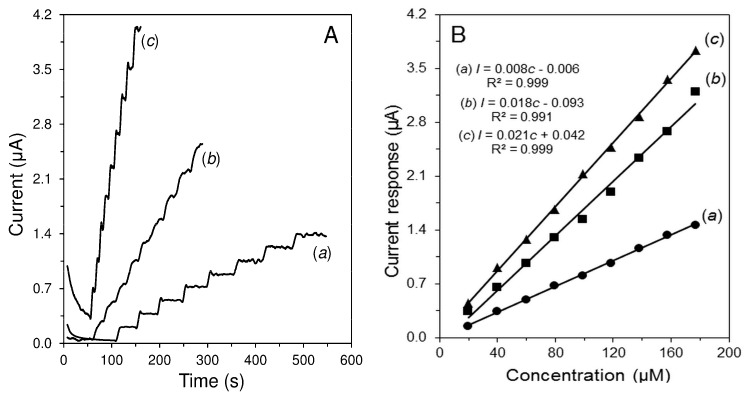
Comparison of (**A**): amperometric records and (**B**): calibration curves, obtained at bare GCE (***a***), GCE/MWCNTs/chitosan (***b***), and GCE/MWCNTs/Nafion^®^ (***c***) to additions of 20 μL H_2_O_2_ (0.1 M). All measurements were performed in 0.1 M phosphate buffer (PB) of pH 7.0 at potential +0.8 V and speed of stirring 400 rpm.

The effect of MWCNTs on H_2_O_2_ amperometric detection was studied using amperometry in a batch configuration. Nearly three times higher sensitivity was obtained in comparison with bare GCE at +0.8 V. Nafion^®^ is better than chitosan for the covering of the electrode as it did not cause a significant decrease in the current compared to the GCE/MWCNTs. Moreover, the obtained amperograms were well-shaped (less noisy) when the electrode was covered with Nafion^®^ (Figure 1). Thus, GCE/MWCNT/Nafion^®^ was selected as the optimum amperometric transducer. Due to its physicochemical properties Nafion^®^ has been considered as a promising candidate for covering the electrode [28].

### 2.2. Effect of Redox Mediator

**Figure 2:**
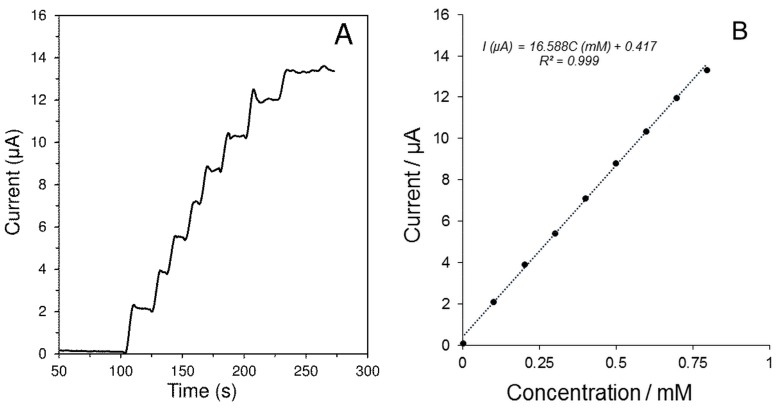
Amperometric records (**A**) and appropriate calibration curve (**B**), obtained at GCE/MWCNTs-RuO_2_/Nafion^®^ to additions of 10 μL H_2_O_2_ (0.1 M). The measurements were performed in 0.1 M PB of pH 7.0 at potential +0.4 V and speed of stirring 400 rpm.

Without using the mediator, the amperometric determination of H_2_O_2_ (product of Glc enzymatic oxidation) occurred at high potential values, around +0.8 V [29]. At this potential value, the interference by species, which can be electrochemically oxidized, is likely to occur. Hence, RuO_2_ was included into the biosensor as the mediator. As can be observed from the cyclic voltammograms shown in Figure S1 (Supplementary Materials) in the presence of RuO_2_, the oxidation peak of H_2_O_2_ shifts to the less positive potentials. In addition, the current signal was also substantially increased in the presence of RuO_2_. It was found that 5% RuO_2_ (w/w) content in MWCNTs dispersion is the optimum for H_2_O_2_ detection at +0.4 V. The chronoamperogram and the related calibration curve of the H_2_O_2_ oxidation on GCE/MWCNTs-R_u_O_2_/Nafion^®^ is also presented in Figure 2. The increased sensitivity can be realized by comparing the slope of the calibration curve of GCE/MWCNTs/Nafion^®^ with that of GCE/MWCNTs-RuO_2_/Nafion^®^. Thus, the determination of Glc can be carried out by the developed GCE/MWCNTs-RuO_2_/GOx/Nafion^®^ at +0.4 V. Concerning the presence of RuO_2_ as the redox mediator, the developed GOx biosensor can be classified into the second generation of biosensors [30].

### 2.3. Characterisation of Biosensor Surface

**Figure 3:**
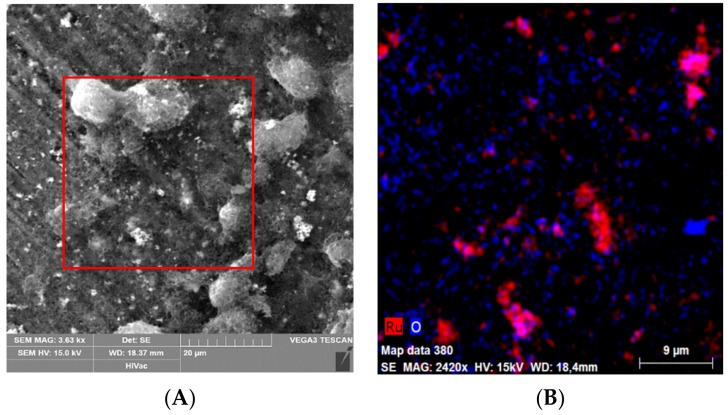
Scanning electron microscopy (**A**) and elemental mapping (**B**) of GCE/MWCNTs-RuO_2_ surface. Both images were taken at the same site.

MWCNTs are defined as electrical conductive materials with large specific surface areas that are widely utilized in amperometric biosensors development [31]. The scanning electron microscopy (SEM) represents a routine tool for characterisation of electrode surfaces morphology. It was confirmed that MWCNTs are not homogeneously spread over the surface of GCE [32] because they create irregular skeins of various sizes with multifaceted range of interconnection (Figure 3A). Moreover, an elemental mapping of GCE/MWCNTs surface showed that RuO_2_ is a redox mediator located in clusters (Figure 3B) as well.

In order to observe the topographic information with a high resolution other techniques such as surface plasmon resonance (SPR) [33,34], electrochemical scanning tunneling microscopy (ESTM) [35], scanning electrochemical microscopy (SECM) [36], and atomic force microscopy (AFM) [37] can be used. As a suitable microscopic technique used for three-dimensional imaging of electrode surface, AFM was applied to collect the complementary data in addition to those obtained by SEM.

**Figure 4:**
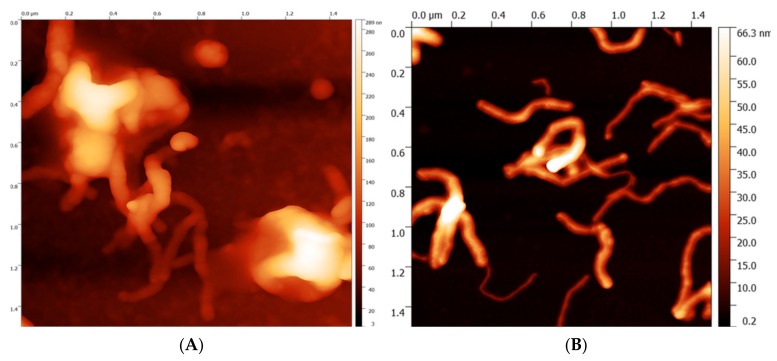
AFM images of (**A**): GCE/MWCNTs/GOx surface. (**B**): GCE/MWCNTs.

As shown in Figure 4A, the molecules of GOx, which are characterized by a molecular weight of 160 kDa are randomly distributed over the electrode surface. The random distribution of GOx might be caused by the drying of the water solvent during adsorption of the enzyme. Moreover, AFM showed that non-specific aggregates of enzymes were created on the GCE/MWCNTs surface. It is necessary to realize that surface of GCE/MWCNTs is not smoothed and rather resembles mountains. Therefore, enzyme molecules accumulated in incurred hollows. The presence of GOx on the electrode surface can also be confirmed by comparing Figure 4A with Figure 4B where only the dispersion of MWCNT was dropped on the electrode (not GOx). The fibrous structure of MWCNTs is illustrated in Figure 4B.

### 2.4. Effect of Stirring Rate

In the amperometry with batch configuration, the transport of the analyte Glc is enhanced by stirring of the magnetic bar. Therefore, the speed of stirring can affect significantly the response in the closed dynamic system. Dependency of current response on the speed of stirring was investigated from 200 to 600 rpm (see Supplementary Materials, Figure S2). The current response increased with the speed of the stirring till 400 rpm. At higher values than 400 rpm, no significant increase of the current response was observed. Thus, the value of 400 rpm was logically chosen as optimal.

### 2.5. Amount of Enzyme Incorporated in Polymer

The current response is influenced by the enzyme amount incorporated in the polymer [38]. The amount of enzyme also affects the polymer properties such as porosity, enzyme retention capacity, polymer adhesion to the electrode surface (to MWCNTs), and mechanical stability [39,40]. The amount of GOx in Nafion^®^ layer was varied from 5 to 25 μg to study its effect on the biosensor performance. As expected, current signal increased with increasing the amount of GOx in the polymer layer up to 20 μg, but no significant current increase was observed when a higher amount of the enzyme was dropped on the electrode (Figure S3). Therefore, the amount of 20 μg was chosen as the optimum to obtain a high current signal.

### 2.6. Effect of the Applied Potential

The working potential plays the most important role in the biosensor function as it has to be kept constant during the analysis [41]. As already mentioned above, the optimum potential of +0.8 V could be applied on GCE/MWCNTs/GOx/Nafion^®^, if the standard aqueous solution of heavy metals is to be analyzed. In this case, a presence of any redox mediator is not necessary. However, if the developed biosensor is to be used for determination of Glc in real samples, a redox mediator [42] must be included to shift the detection potential to lower values. Any interference of accompanying substances is not assumed at potentials close to 0.0 V. Therefore, RuO_2_ was used as a redox mediator in the developed biosensor.

A dependency of current response on detection potential for GCE/MWCNTs/GOx-RuO_2_/Nafion^®^ was investigated from 0.0 to +0.8 V (Figure S4). It was observed that the current response increased by applying higher values of potentials up to +0.4 V. The detection potential of +0.4 V was therefore selected as optimum.

### 2.7. Analytical Performance of Proposed GOx Biosensor

**Figure 5:**
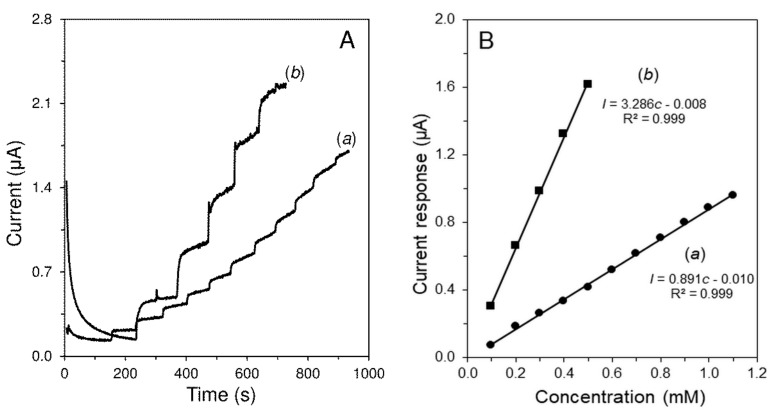
Typical amperometric responses (**A**) and corresponding calibration curves (**B**) of the CPE/RuO_2_/GOx (***a***) and GCE/MWCNTs-RuO_2_/GOx/Nafion^®^ (***b***) to additions of 110 μM Glc. Measured in 0.1 M PB of pH 7.0 at potential +0.4 V and speed of stirring 400 rpm.

When the baseline shown in Figure 1 is compared to that of Figure 5, a drifting of baseline is evident for the sensors containing GOx enzyme in their recognition layers. Waiting for the baseline stabilization before adding of substrate did not help to decrease the drifting of baseline. From Figure 5, it should be clear that the drifting of baseline is steeper after each addition of Glc. This phenomenon could be probably attributed to the analyte transport across the Nafion^®^ layer and/or irregular flow of the working medium. The latter results from the comparison of baselines obtained for modified CPE and GCE covered by thin layer of MWCNTs with polymer. It seems that the drifting of baseline increases with the complexity of biorecognition layer. However, this phenomenon does not have any effect on evaluation of current responses because the differences of current levels (the current jump) were used for the plotting of calibration curves. For analytical applications, it is necessary to obtain reproducible current jumps for certain substrate concentration, and this has been achieved in this work.

Figure 5 shows typical amperograms obtained at CPE/RuO_2_/GOx (bulk modified) and the developed GCE/MWCNTs-RuO_2_/GOx/Nafion^®^ in analysis of Glc. A relatively strong drifting of current baselines in both cases was found. This phenomenon is probably caused by the presence of GOx because any drifting was not observed during comparison of amperometric transducers, as shown in Figure 1. Fortunately, important subtraction of current responses did not worsen dramatically.

At first sight, it is evident that a noticeable increase in the sensitivity has been achieved using carbon nanotubes. A linear dependencies of current response (*I*) on Glc concentration (*c*) are described by following equations *I* = 0.891*c* − 0.010 with the correlation coefficient (R^2^) 0.999 for CPE/RuO_2_/GOx and *I* = 3.286*c*− 0.008 with R^2^ = 0.999 for GCE/MWCNTs-RuO_2_/GOx/Nafion^®^ for a concentration range from 0.1 to 1.0 mM Glc. Limits of detection (LOD) and quantification (LOQ) were calculated as 3.3 *s*/*k* and 10 *s*/*k*, respectively, for both GOx biosensors. Where *s* represents the standard deviation for 5 repetitions and *k* is the slope (sensitivity) of the corresponding equation. LOD of 28.9 μM and LOQ of 87.7 μM Glc were obtained at CPE/RuO_2_/GOx. On the other hand, significantly better analytical parameters were achieved at GCE/MWCNTs-RuO_2_/GOx/Nafion^®^, namely LOD of 17.4 μM and LOQ of 52.7 μM Glc. Therefore, concerning the blood Glc level (3.9 mM ≥ [Glc] ≤ 7.1 mM) the proposed GOx biosensor could be utilized in the clinical analysis [43]. The analytical figures of merit of the developed sensor for determination of H_2_O_2_ and Glc are presented in Table 1.

The nature of the inhibition of the enzyme activity by the heavy metals was also investigated. Two calibration curves of the Glc at the developed sensor were plotted in the presence of a given concentration of Hg^2+^ and without Hg^2+^ in the solution. The noncompetitive inhibition of mercury was confirmed using Michaelis Menten model (see Supplementary Materials, Figure S5). Since the values of *K_M_* (Michaelis–Menten constant) were nearly identical, but *V*_max_ (maximum initial velocity) was decreased in presence of the Hg^2+^, the inhibition effect of heavy metals is supposed to be noncompetitive [44].

**Figure 6:**
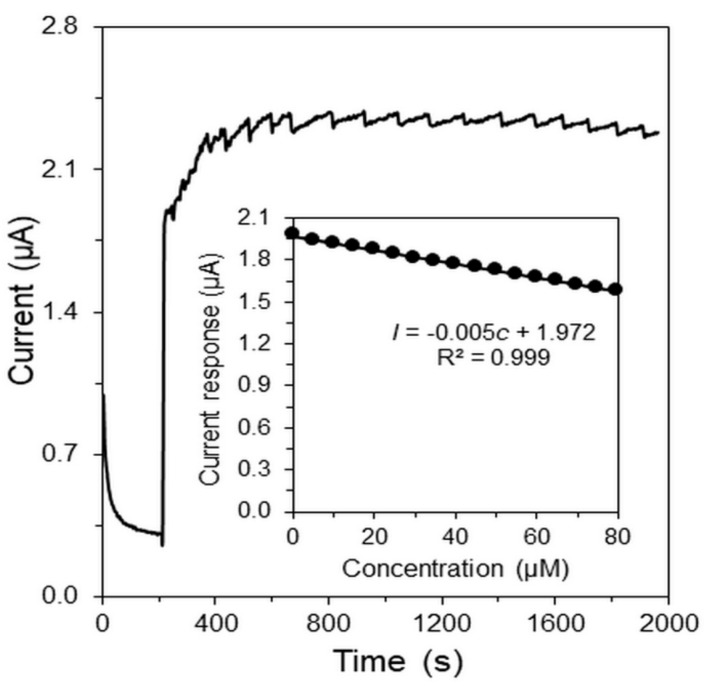
Amperometric response of a GCE/MWCNTs-RuO_2_/GOx/Nafion^®^ to the first addition of 550 μM glucose and subsequent additions of 5.0 μM mercury(II). The measurement was performed in 0.1 M PB of pH 7.0 at potential +0.4 V and speed of stirring 400 rpm.

The current work was aimed to investigate the monitoring of enzyme activity inhibition by heavy metals. A few works focusing on indirect determination of mercury [45,46] or other heavy metals [47,48] by inhibition of GOx immobilized on different amperometric transducers have already been published. Figure 6 shows a typical amperogram obtained at GCE/MWCNTs-RuO_2_/GOx/Nafion^®^ for 550 μM Glc (the first addition) with subsequent addition of 5 µM mercury(II) cation. Corresponding calibration curve for concertation range from 5 to 80 μM of Hg^2+^ is also presented (insert Figure 6). Theoretical values of LOD of 1.05 μM and LOQ of 3.18 μM Hg^2+^ was determined. The comparison between the developed biosensors for indirect determination of Hg^2+^ is presented in Table 2. However, the developed enzymatic biosensor is accompanied by several disadvantages such as sophisticate construction of the biosensors, short life time of biosensors and risk of interfering substances. From the analytical point of view, the voltammetric methods including a stripping step [49,50,51,52,53,54,55,56,57,58] are preferable to use due to higher sensitivity. Overall, the amperometric enzymatic biosensors represent bio-analytical devices that are more suitable for toxicity study of heavy metals and other poisons.

### 2.8. Study of the Inhibitory Effects of Heavy Metals

Several heavy metals cations (Ag^+^, Cd^2+^, Cu^2+^, and Hg^2+^) were selected to study their inhibition effect on GOx enzyme obtained from *Aspergillus niger* (EC 1.1.3.4). In principle, it is possible to compare the effect of heavy metals on the basis of a decrease in current response for a certain Glc concentration. In general, it can be assumed that the inhibitory effect of heavy metals could be studied for other enzymes using similar protocol. It should be noted that the enzyme-based biosensor is used to study the inhibition of enzyme activity by heavy metals. Therefore, some studies have demonstrated that GOx biosensors can be completely regenerated by the addition of ethylenediaminetetraacetic acid (EDTA) [43,46].

Despite the insufficient reproducibility (7.4% RSD) of five freshly prepared GOx biosensors, the study of heavy metals effect can be realized because the ratios between the current response of substrate and the decrease in current caused by the presence of the heavy metal are compared. A comparison of the obtained results is presented in Table 3. Moreover, the values of response time, which is defined as the time duration from the analyte addition to the baseline stabilization, are represented in Table 3.

**Figure 7:**
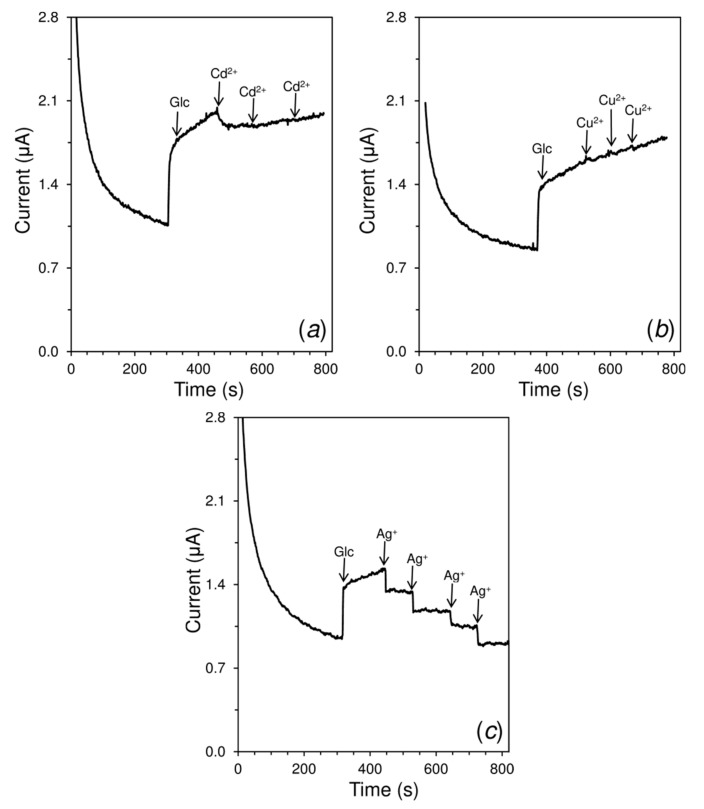
Amperometric responses of 200 μM glucose with consecutive additions of 50 μM Cd^2+^ (**a**), 50 μM Cu^2+^ (**b**), 50 μM Ag^+^ (**c**) at GCE/MWCNTs-RuO_2_/GOx/Nafion^®^. All measurements were performed in 0.1 M PB of pH 7.0 at potential +0.4 V and speed of stirring 400 rpm.

Figure 7 presents a comparison of amperograms obtained for inhibition of GOx enzyme activity by Cu^2+^, Cd^2+^, and Ag^+^. From all tested heavy metals, Cu^2+^ does not have any significant inhibitory effect on GOx catalytic activity. The inhibitory effect of Ag^+^ was two time lower than that of the Hg^2+^. In fact, Ag^+^ inhibits the activity of enzymes that are involved in bacterial cells division and thus significantly slow their proliferation [64].

Except of the Cd^2+^, the shapes of the amperograms are the same for all the studied heavy metal ions. A slower inhibition of GOx catalytic activity can be attributed to Cd^2+^ compared to Hg^2+^ and Ag^+^ due to its longest response time. For the initial concentration of 200 µM of Glc, an evident decrease of current response was only obtained for the first addition of 50 µM Cd^2+^. The subsequent additions did not cause any reduction in current magnitude. Thus, if cadmium(II) is indirectly determined using GOx biosensors, a very short linear range is expected in comparison with other heavy metals. An amperometric GOx biosensor modified with cobalt or copper hexacyanoferrate was also developed for monitoring heavy metals and similar behavior of cadmium(II) was also observed. The linear range for cadmium(II) determination with both types of GOx biosensors was 1.5–6.0 µM in the presence of 300 µM Glc [23].

Due to the high GOx enzyme activity inhibition by heavy metals, the development of GOx biosensors for selective indirect determination of a given heavy metal remains still a challenge in the field of bioelectroanalysis, especially in environmental analysis. It seems that amperometric biosensors could be used in the toxicity study of other poisons.

Herein, it is necessary to mention that an adsorption of heavy metals cations from aqueous solutions on Nafion^®^ 117 membrane have been already studied by Malaysia scientists [58]. They found that this phenomenon is caused by an electrostatic interaction between sulfonic groups of Nafion^®^ (anions) and heavy metals (cations). An accumulation of heavy metals on the membrane layer could increase the exposition of GOx enzyme to a higher concentration of heavy metals than what is actually added to the solution. These authors also tested Cu^2+^ which showed the highest affinity in the comparison with Co^2+^, Ni^2+^, Pb^2+^, and Ag^+^ [58]. Moreover, Cu^2+^ cations did not cause any significant decreases in current response.

An amperometric experiments were carried out to find out the effect of the Nafion^®^ on the function of the developed sensor. Two biosensors (CPE/RuO_2_/GOx and CPE/RuO_2_/GOx covared by thin layer of Nafion^®^) were compared in measurements of glucose calibration when Hg^2+^ was presented in both cases (see Figure S6). It was found that Nafion^®^ did not have any significant effect on Hg^2+^ accumulation. From Lineweaver–Burk plots [65], values of K_M_ were calculated, namely 8.41 mM for CPE/RuO_2_/GOx and 8.25 mM glucose for CPE/RuO_2_/GOx/Nafion^®^. A slight decreasing of *V*_max_ was observed in case of using Nafion^®^. It seems that Nafion^®^ probably only partially protects the enzyme against heavy metals.

## 3. Materials and Methods

### 3.1. Chemicals

Glucose oxidase (GOx) obtained from *Aspergillus niger* ≥100,000 U·g^−1^ solid (EC 1.1.3.4), β-d-glucose, ruthenium oxide used as mediator, hydrogen peroxide (H_2_O_2_), multi-walled carbon nanotubes (MWCNTs), Nafion^®^, acetic acid (AA), chitosan (~50 kDa) and N,N-dimethylformamide (DMF), ethanol (99.98%), nitric acid (70%), ammonia solution (25%), and paraffin oil were purchased from Sigma-Aldrich (St. Louis, MO, USA). Inorganic salts of p.a. grade (CuSO_4_·5H_2_O, AgNO_3_, Hg(NO_3_)_2_·H_2_O, and Cd(NO_3_)_2_·4H_2_O) from Lach-Ner, s.r.o. (Neratovice, Czech Republic) were dissolved in deionized water for preparation of 0.01 M heavy metals stock solutions. This water (18.2 MΩ·cm) had been first double distilled by an Aqua Osmotic 02 from Aqua Osmotic (Tišnov, Czech Republic) and then deionized using a Millipore RG (Milli-Q water, Millipore Corp., Billerica, MA, USA). PB was prepared by mixing 0.2 M NaH_2_PO_4_ and 0.2 M Na_2_HPO_4_ (Sigma-Aldrich, St. Louis, MO, USA) in 1:1 volume ratio. The required pH value then was achieved by the appropriate addition of 1 M NaOH. The expanded graphite was purchased from Graphite Týn Ltd. (Týn nad Vltavou, Czech Republic).

### 3.2. Apparatus

The imaging of the developed GCE/MWCNTs amperometric transducer surface was carried out by the scanning electron microscope, Vega3 SB from TESCAN Brno, s.r.o. (Brno, Czech Republic). Furthermore, energy dispersive spectroscopy (EDS) was utilized for elemental mapping of the transducer surface. The imaging was carried out by applying a potential of 15 kV at 18.4 mm working distance. Atomic force microscopy (AFM) for the electrode surface characterization of the developed GCE/MWCNTs-RuO_2_/GOx/Nafion^®^ was carried out at dimension FastScan Bio from Bruker (Billerica, MA, USA) operating with Gwydion 2.52 for data visualization [66]. An Autolab electrochemical analyser model "PGSTAT-101" running by Nova 2.1 software from Metrohm Autolab (Utrecht, The Netherlands) was used to execute the amperometric experiments in a batch configuration. The conventional three-electrode system was used. A platinum wire as the counter electrode, Ag/AgCl 3 M KCl as the reference electrode and GOx biosensor as the working electrode were served. All the measurements were carried out in one-compartment voltammetric cells (10–20 mL) at conditioned room temperature (23 ± 1 °C). The pH measurements were performed using a pH meter Model Sentix 81 from WTW (Weilheim, Germany) with a combined electrode (glass electrode-Ag/AgCl (3 M KCl) reference electrode) with an accuracy of pH ± 0.05.

### 3.3. Preparation of Working Electrodes

The GCE; No. 6.1204.110 with a diameter 3 mm was purchased from Metrohm (Prague, Czech Republic). It was polished on a polishing pad using alumina powder (0.3 and 0.05 μm) for 1 min. followed by sonication in deionized water for 1 min and in ethanol for another 1 min. Then, it was subjected to the various modifications to prepare different electrodes, as described below.

To improve the sensitivity of GOx biosensor, it was necessary to increase the active surface area of amperometric transducer. Therefore, the GCE surface was covered by a thin layer of MWCNTs (GCE/MWCNTs). In this case, a mixture of 2.0 mg of MWCNT and 0.1 mg RuO_2_ was dispersed in 1.000 mL of DMF and put in ultrasonic bath (Singen, Germany) for 1 h with applied frequency 37 kHz, 10 µL of the dispersion was then dropped onto GCE surface and it was left to be dried at room. The content of 5% RuO_2_ (w/w) was incorporated into MWCNT due to better amperometric detection of hydrogen peroxide produced by biocatalytic oxidation [58].

Two different polymers (chitosan and Nafion^®^) were tested for GOx immobilization. First GOx enzyme had to be directly adsorbed onto GCE/MWCNTs. A desired volume of the GOx solution (2.0 mg·mL^−1^ water solution) was dropped onto the prepared GCE/MWCNTs. When dried the electrode was further covered by the desired polymer.

The chitosan was prepared as 1% chitosan solution dispersed in 1% acetic acid containing 1.0 mL of HNO_3_ in 1:1 volume ratio. Then 10 µL of the prepared solution was dropped on the electrode surface. Electrodes that were prepared in this way were stored in the refrigerator at 4 °C overnight. The Nafion^®^ had to be neutralized by addition of 8% ammonia solution due to presence of sulfonic groups in the chemical structure [67]. After that, 10 µL of 1% Nafion^®^ (v/v) was injected onto electrode surface and left to be air-dried under laboratory conditions. If no inhibition of heavy metals was measured, GOx biosensor (GCE/MWCNTs-RuO_2_/GOx/Nafion^®^) was stored in a refrigerator at 4 °C.

Additionally, a simple GOx sensor based on bulk modified carbon paste electrode (CPE/RuO_2_/GOx) was prepared [68]. The modification was carried out in a ceramic mortar by homo-genizing 0.3 g graphite powder with 90 mg paraffin oil, 20 mg RuO_2_, and 45 mg glucose oxidase (GOx) for 20 min. The prepared paste was then filled into the Teflon^®^ piston-like electrode holder (diameter 3 mm) [69]. The electrode surface had to be renewed after each analysis, by squeezing out of small portion of the carbon paste filling and polishing against a dry filter paper to achieve the spilling of the present GOx.

### 3.4. Procedure

Amperometry in a batch configuration at interval time 1.0 s, constant working potential +0.4 V and stirring speed 400 rpm was used. Due to the optimum biocatalytic activity of GOx [41], all amperometric measurements were performed in 0.1 M PB of pH 7.0. The inhibition effect of the heavy metals (expect Hg^2+^) on the developed GOx biosensor was investigated by the addition of 50 µL of their standard solution (0.01 M) into 10 mL of the measuring solution containing the Glc. In the case of Hg^2+^ the 5 µL of the stock solution was added to the 10 mL of the measuring solution. The added volume of the heavy metals was taken into account in plotting the calibration curves.

## 4. Conclusions

An enzymatic Glc biosensor was developed which was successfully applied for determination of H_2_O_2_ and Glc. The LOD was calculated for to be 17.4 µM for Glc. Furthermore, through this electroanalytical study, the toxicity effect of the selected heavy metals cations on the GOx enzyme from *Aspergillus niger* (EC 1.1.3.4) was compared using a new amperometric biosensor. The developed biosensor can be applied for rapid and accurate monitoring of enzyme activity in industry and medical laboratories for preliminary tests. Unlike the other heavy metals studied herein, it was found that Cu^2+^ has a negligible inhibition effect on GOx catalytic activity. The mercury(II) had the highest inhibition effect among all selected heavy metals. The inhibitory effect of Ag^+^, and Cd^2+^ were also shown by the developed biosensor. In future, it can be assumed that non-competitive inhibition of various poisons (not only heavy metals) on the activity of other enzymes could also be studied using the same protocol. The developed sensor can be utilized in ore mining or screening wastewater of factories.

## Figures and Tables

**Table 1:** The analytical parameters obtained for the calibration of H_2_O_2_ (at GCE/MWCNTs-RuO_2_/Nafion^®^) and Glc at (GCE/MWCNTs-RuO_2_/GOx/Nafion^®^).

Parameter	H_2_O_2_	Glc
Peak potential (V)	+0.40	+0.40
Linearity range (µM)	10–800	100–800
Slope (µA∙µM^−1^)	0.017 ± 0.005	0.003 ± 0.0002
Correlation coefficient (*r*)	0.999 ± 0.001	0.999 ± 0.001
^1^ LOD (µM)	7.5	17.4
LOQ (µM)	25.0	52.7
RSD% (*n* = 3)	4.2	3.5
^2^ Confidence interval (µM)	1.3	1.1

^1^ Obtained with 3.3 *s*/*k* (*s*: standard deviation, *k*: slope of the calibration curve); ^2^ confidence interval of 95% = $\frac{ts}{\surd n}$ (*s*: standard deviation, *t:* critical value for 3 repetitions: 4.30). RSD (%): relative standard deviation.

**Table 2:** Some of enzyme based electrochemical biosensors developed for mercury determination.

Electrode Material	Electrochemical Technique	Enzyme	Linear Range	LOD	Ref.
SPCEs	Amperometry	Urs	0.37–4.99 µM	0.31 µM	[59]
ISFET	Potentiometry, Conductometry	AChE, BCHE	10–50 µM	10 µM	[60]
ZnO-NRs	Potentiometry	GOx	50 nM–20 mM	0.5 nM	[61]
PtE	Amperometry	GOx	5–180 µM	2.5 µM	[62]
CPE	Amperometry	GOx	10–160 µM	2.5 µM	[63]
GCE/MWCNT-RuO_2_/Nafion^®^	Amperometry	GOx	5–80 µM	1.0 µM	This work

AChE; Acetylcholinesterase, BCHE; Butyrylcholinesterase, PtE; platinum electrode, NRS; nanorods, SPCE; screen-printed carbon electrode and Urs; urease.

**Table 3:** Inhibitory effect of selected heavy metals to GOx.

Heavy Metal	^1^ Relative Inhibition (%)	Response Time (s)
Cadmium(II)	34.2	37
Copper(II)	8.4	16
Mercury(II)	100	10
Silver(I)	48.6	9

^1^ The relative inhibition was calculated for 550 μM glucose and 100 μM of heavy metal solutions. All values are shown as averages of minimally three measurements.

## Supplementary Materials

The following are available online at https://www.mdpi.com/1424-8220/19/13/2939/s1, Figure S1: Cyclic voltammograms of 0.1 M PB (blank) pH 7.0 and in presence of the 5 × 10−3 M of the H2O2 obtained at different electrodes and at scan rate was 50 mV·s−1. Figure S2: Effect of stirring rate on oxidation current response of 50 μM hydrogen peroxide. Results were obtained from amperometric measurements (always for 5 repetitions) in the batch configuration at GCE/MWCNTs/Nafion® in 0.1 M PB of pH 7.0 at potential +0.8 V. Figure S3: Effect of amount of the glucose oxidase from Aspergillus niger (EC 1.1.3.4) embedded in Nafion® membrane on current response of 200 μM glucose. Results were obtained from amperometric measurements (always for 5 repetitions) in the batch configuration at GCE/MWCNTs/GOx/Nafion® in 0.1 M PB of pH 7.0 at potential +0.8 V and stirring rate 400 rpm. Figure S4: Effect of applied potential on current response of 150 μM glucose. Results were obtained from amperometric measurements (always for 5 repetitions) in the batch configuration at GCE/MWCNTs/GOx-RuO2/Nafion® in 0.1 M PB of pH 7.0 at potential +0.4 V and stirring rate 400 rpm. Figure S5: Typical amperograms with corresponding calibration curves of glucose without (solid; a) and with content of 250 μM Hg2+ (dashed line; b) obtained at CPE/RuO2/GOx in 0.1 M PB of pH 7.0 at potential +0.8 V and speed of stirring 400 rpm (A). Lineweaver–Burk plot confirmed noncompetitive inhibition of mercury (B). Figure S6: Typical amperograms with corresponding calibration curves of glucose with content of 250 μM Hg^2+^ obtained at CPE/RuO_2_/GOx (dashed; b) and CPE/RuO_2_/GOx/Nafion® (dotted line; c) in 0.1 M PB of pH 7.0 at potential +0.8 V and speed of stirring 400 rpm (C). Comparison of appropriate Lineweaver–Burk plots (D).
