# Supplementary material for: Application of the Enzymatic Electrochemical Biosensors for Monitoring Non-Competitive Inhibition of Enzyme Activity by Heavy Metals
Source: Sensors (Basel). 2019 Jul 3;19(13):2939. doi: 10.3390/s19132939 (PMC6651500; doi:10.3390/s19132939)
Supplement: Supplementary file 1 [file sensors-19-02939-s001.pdf]

Supplementary material

Application of the Enzymatic Electrochemical Biosensors for Monitoring Non-Competitive Inhibition of Enzyme Activity by Heavy Metals

Amir M. Ashrafi [1,2,3], Milan Sýs [3], Eliška Sedláčková [1,2], Amir Shaaban Farag [3], Vojtěch Adam [1,2], Jan Pribyl [4], and Lukáš Richtera [1,2*]

1 Department of Chemistry and Biochemistry, Mendel University in Brno, CZ-613 00 Brno, Czech Republic; ashrafi@mendelu.cz (A.M.A.); eliska.sedlackova@mendelu.cz (E.S.); vojtech.adam@mendelu.cz (V.A.); richtera@mendelu.cz (L.R.)

2 Central European Institute of Technology, Brno University of Technology, 612 00 Brno, Czech Republic; ashrafi@mendelu.cz (A.M.A.); eliska.sedlackova@mendelu.cz (E.S.); vojtech.adam@mendelu.cz (V.A.); richtera@mendelu.cz (L.R.)

3 Department of Analytical Chemistry, Faculty of Chemical Technology, University of Pardubice, Studentská 573, 532 10 Pardubice, Czech Republic; milan.sys@upce.cz (M.S.); st56093@student.upce.cz (A.S.F.)

4 CEITEC MU, Masaryk University, Brno, Czech Republic; pribyl@nanobio.cz (J.P.)

* Correspondence: richtera@mendelu.cz; Tel.: +420-545-133-311

Received: 27 May 2019; Accepted: 1 July 2019; Published: date

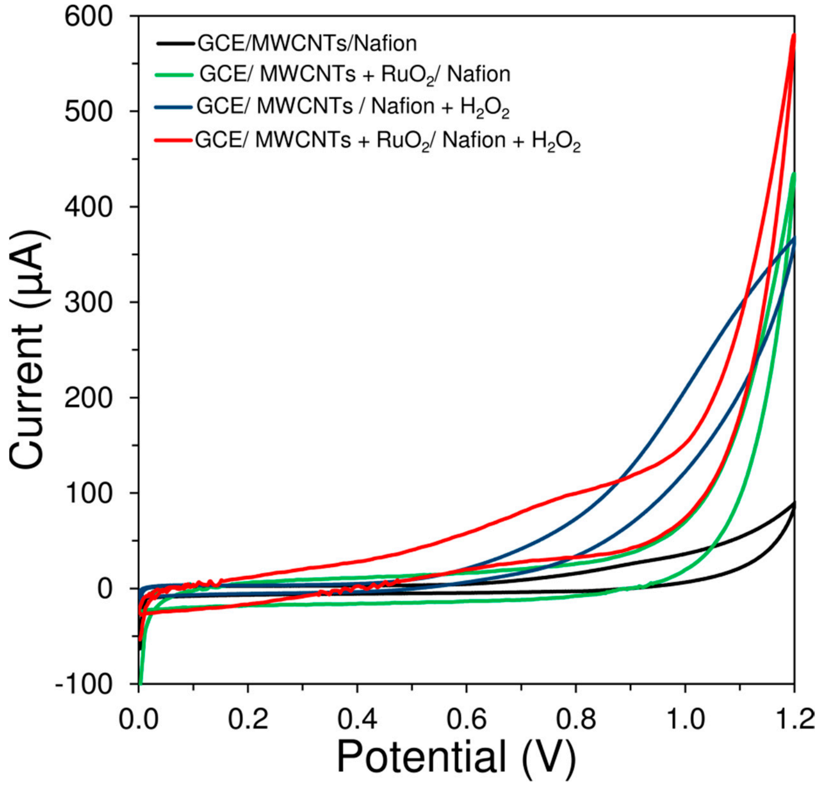

Figure S1: Cyclic voltammograms of 0.1 M phosphate buffer (blank) pH 7.0 and in presence of the $5{\times}10^{-3}$ M of the H_2O_2 obtained at different electrodes and at scan rate was 50 mV s^{-1}.

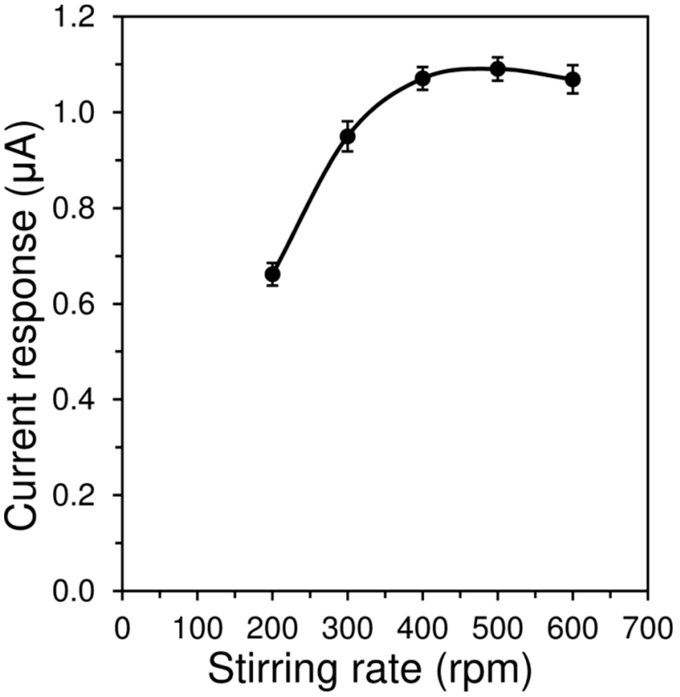

Figure S2. Effect of stirring rate on oxidation current response of 50 μM hydrogen peroxide. Results were obtained from amperometric measurements (always for 5 repetitions) in the batch configuration at GCE/MWCNTs/Nafion® in 0.1 M phosphate buffer of pH 7.0 at potential +0.8 V.

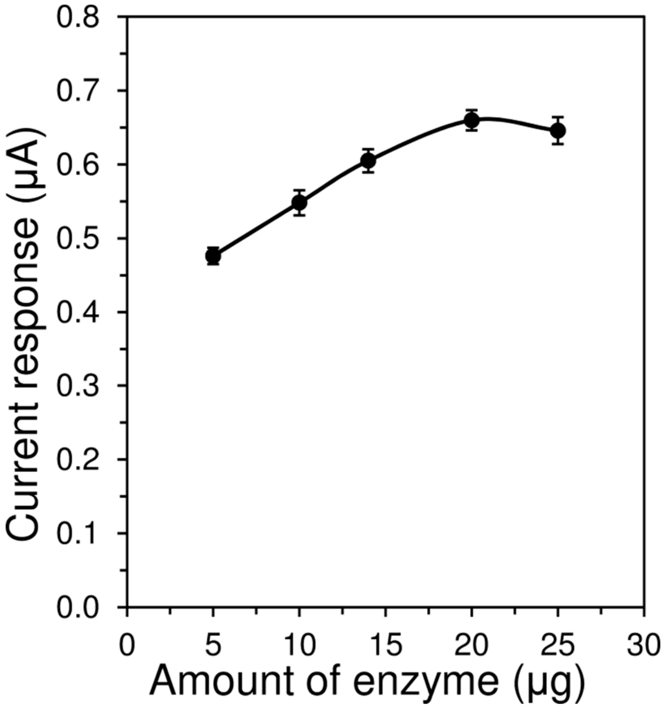

Figure S3. Effect of amount of the glucose oxidase from *Aspergillus niger* (EC 1.1.3.4) embedded in Nafion® membrane on current response of 200 μM glucose. Results were obtained from amperometric measurements (always for 5 repetitions) in the batch configuration at GCE/MWCNTs/GOx/Nafion® in 0.1 M phosphate buffer of pH 7.0 at potential +0.8 V and stirring rate 400 rpm.

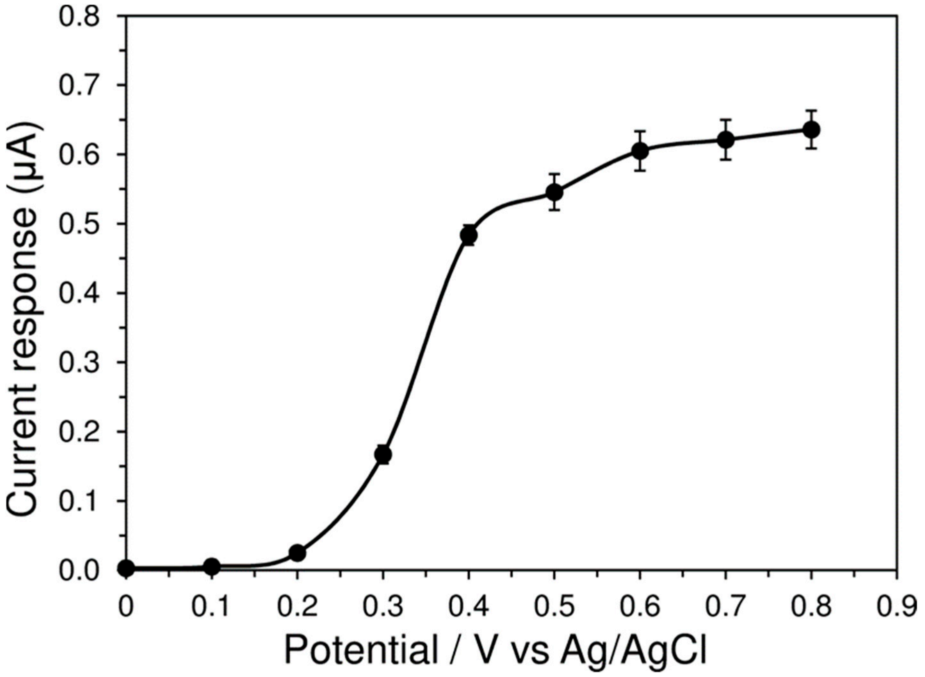

Figure S4. Effect of applied potential on current response of 150 µM glucose. Results were obtained from amperometric measurements (always for 5 repetitions) in the batch configuration at GCE/MWCNTs/GOx-RuO_2/Nafion® in 0.1 M phosphate buffer of pH 7.0 at potential +0.4 V and stirring rate 400 rpm.

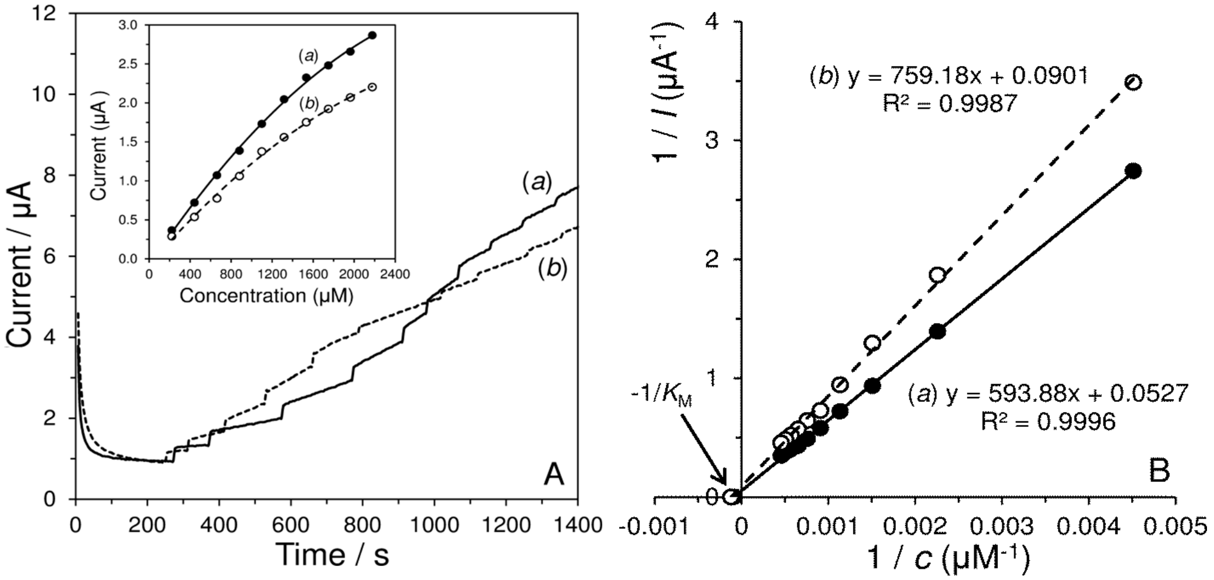

Figure S5. Typical amperograms with corresponding calibration curves of glucose without (solid; *a*) and with content of 250 µM Hg^{2+} (dashed line; *b*) obtained at CPE/RuO_2/GOx in 0.1 M phosphate buffer of pH 7.0 at potential +0.8 V and speed of stirring 400 rpm (A). Lineweaver–Burk plot confirmed noncompetitive inhibition of mercury (B).

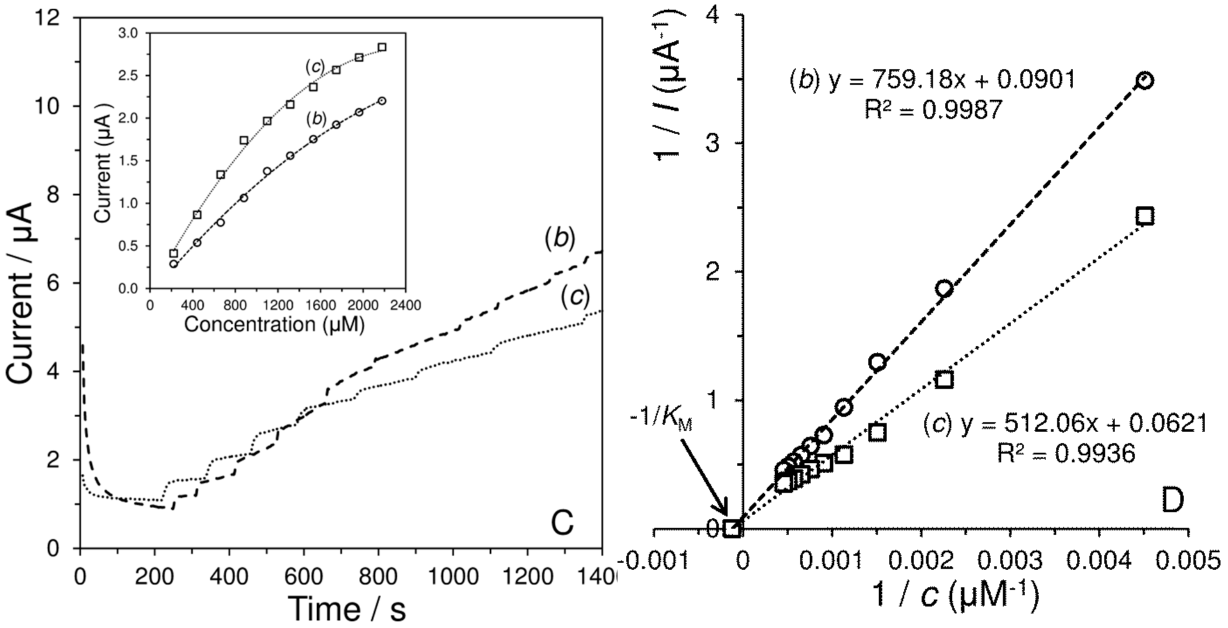

Figure S6. Typical amperograms with corresponding calibration curves of glucose with content of 250 μM Hg^{2+} obtained at CPE/RuO_2/GOx (dashed; *b*) and CPE/RuO_2/GOx/Nafion® (dotted line; *c*) in 0.1 M phosphate buffer of pH 7.0 at potential +0.8 V and speed of stirring 400 rpm (C). Comparison of appropriate Lineweaver–Burk plots (D).
